# Supplementary material for: Aldo-Keto Reductase 1C1 (AKR1C1) as the First Mutated Gene in a Family with Nonsyndromic Primary Lipedema
Source: Int J Mol Sci. 2020 Aug 29;21(17):6264. doi: 10.3390/ijms21176264 (PMC7503355; doi:10.3390/ijms21176264)
Supplement: Supplementary file 1 [file ijms-21-06264-s001.zip › ijms-889202_Supplementary information.docx]

Supplementary Information

Aldo/Keto-Reductase 1C1 (*AKR1C1*) as the first mutated gene in a family with non-syndromic primary Lipedema

Sandro Michelini ^1^, Pietro Chiurazzi ^2^ , Valerio Marino ^3^, Daniele Dell’Orco ^4^, Elena Manara^5^, Mirko Baglivo ^6^, Alessandro Fiorentino ^7^, Paolo Enrico Maltese ^8^, Michele Pinelli ^9^, Karen Louise Herbst ^10^, Astrit Dautay ^11^, and Matteo Bertelli ^12,^*

**Supplementary Table 1**. Variants identified in Family 1 by WES. For each variant, nucleotide and aminoacid change, frequency and ACMG classification are reported. In addition, three tissues in which the expression of that gene is higher are indicated in the GTEX column (Genotype-Tissue Expression).

| **GENE** | **Nucleotide change (hg19)** | **AA change** |  | **Freq in GNOMAD** | **Mutation tolerance** | **ACMG classification** | **Tissue Expression (**[**GTEx**](http://varso.me/go/to?url=https%3A%2F%2Fgtexportal.org%2Fhome&q=voxKjr)**)** |
| --- | --- | --- | --- | --- | --- | --- | --- |
| *NGEF* | NM_001114090.1:c.614C>T | p.Ser205Phe | rs780444133 | 0.00000798 | MI | VUS | Brain Cortex, Frontal Cortex, Basal Ganglia |
| *ADK* | NM_001123.3:c.868A>G | p.Lys307Glu | rs760279818 | 0.0000119 | MT | VUS | EBV transformed lymphocytes, Liver, Esophagus |
| *FBXL7* | NM_001278317.1:c.187G>A | p.Glu63Lys | rs778729469 | 0.0000287 | MT* | VUS | Artery, Ovary, Uterus |
| *PITPNM1* | NM_001130848.1:c.1594G>A | p.Val532Met | rs112955177 | 0.0000442 | MT* | VUS | Brain Cortex, Cerebellum, Frontal Cortex |
| *FAM160B2* | NM_022749.5:c.712G>T | p.Gly238Cys | rs139434422 | 0.000184 | MI | Likely Benign | Pituitary, Thyroid, Prostate |
| *AKR1C1* | NM_001353.5:c.638T>A | p.Leu213Gln | rs372782197 | 0.000207 | MT | Likely Benign | Adipose -subcutaneous, Liver, Heart |
| *FRYL* | NM_015030.1:c.2908A>T | p.Met970Leu | rs202125385 | 0.000522 | MI | Likely benign | EBV transformed lymphocytes, Cultured fibroblasts, Colon |

**Supplementary Table 2**. Single or double linear correlation coefficients of the functional parameters and independent variables identified by the MD descriptors.

| ***R^2^*** | ***SAS NADP+*** | ***SAS hPGS*** | ***IE_P-hP_*** | ***IE_N-hP_*** | ***SAS NADP+ and***  ***SAS hPGS*** | ***IE_P-hP_***  ***and***  ***IE_N-hP_*** |
| --- | --- | --- | --- | --- | --- | --- |
| **log(K_m_)** | 0.03 | 0.01 | 0.04 | 0.13 | 0.03 | 0.26 |
| **log(k_cat_)** | 0.32 | 0.68 | ***0.72*** | 0.11 | 0.69 | 0.77 |
| **log(k_cat_/K_m_)** | 0.04 | 0.18 | 0.48 | 0.04 | 0.18 | **0*.73*** |

**Supplementary Table 3**. Single linear correlation coefficients of the independent variables identified by the MD descriptors.

| **R^2^** | **SAS NADP+** | **SAS hPGS** | **IE_P-hP_** | **IE_N-hP_** |
| --- | --- | --- | --- | --- |
| **SAS NADP+** | 1 | 0.32 | 0.17 | 0.22 |
| **SAS hPGS** | 0.32 | 1 | 0.63 | 0.22 |
| **IE_P-hP_** | 0.17 | 0.63 | 1 | 0.13 |
| **IE_N-hP_** | 0.22 | 0.22 | 0.13 | 1 |

**Supplementary Figure 1**. Root-mean square fluctuation of Cα atoms calculated over 1 µs MD simulations of AKR1C1 WILD-TYPE (black) and L213Q (red). Residues belonging to loops A, B and C are highlighted as in Figure 2.


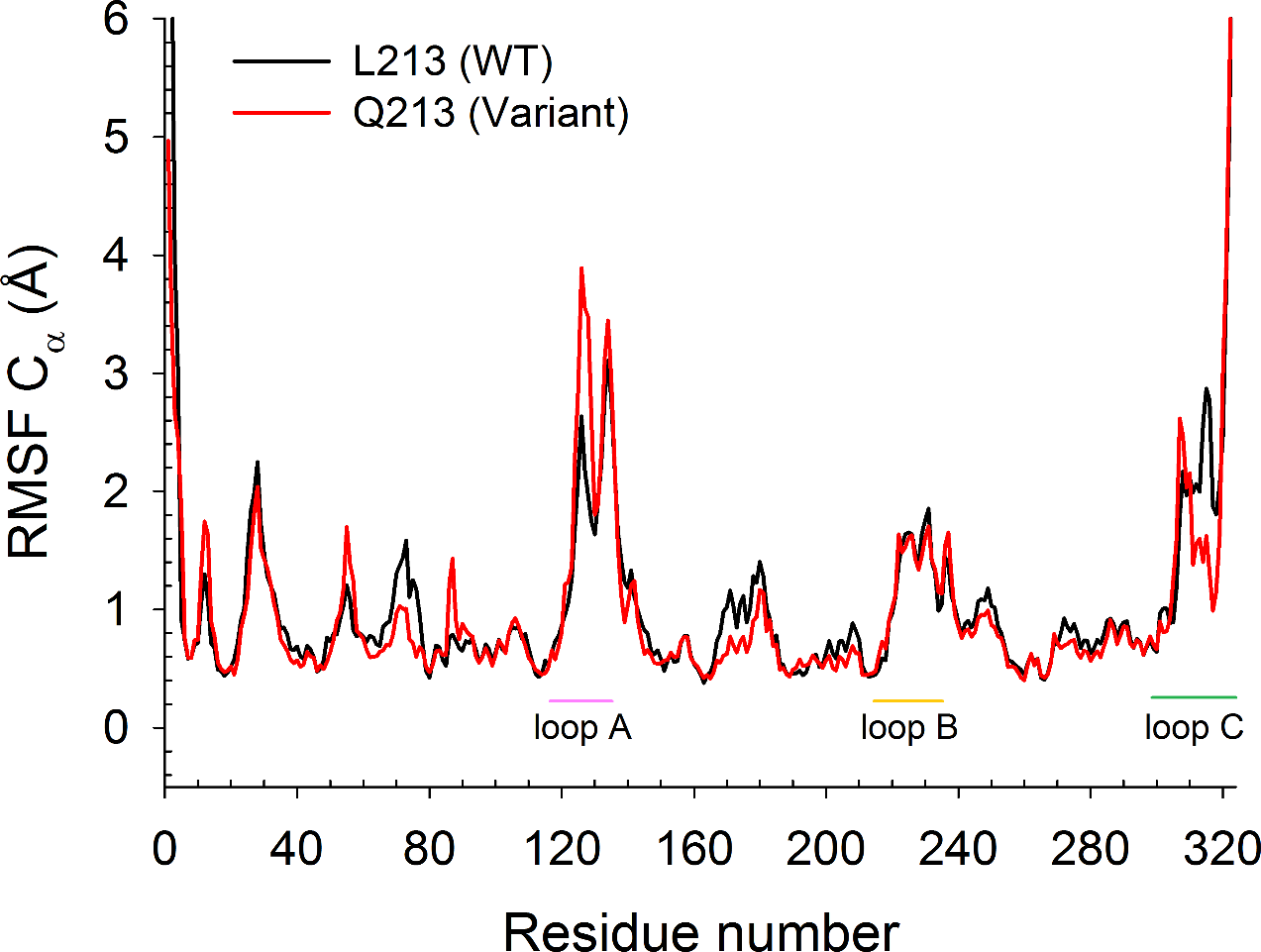


**Supplementary Video S1.** Solvent-accessible surface of AKR1C1 WILD-TYPE. Protein structure is shown as light blue cartoons, the solvent-accessible surface of the protein, NADP+ and hPGS are shown in light blue, orange and blue, respectively.

**Supplementary Video S2.** Solvent-accessible surface of AKR1C1 L213Q. Protein structure is shown as light orange cartoons, the solvent-accessible surface of the protein, NADP+ and hPGS are shown in light orange, orange and blue, respectively.
